# Fibrous dysplasia of the middle nasal turbinate: imaging and clinical significance

**DOI:** 10.1259/bjrcr.20150296

**Published:** 2016-11-02

**Authors:** Venkatraman Bhat, Kanav Kansal, Shri Harsha Krishna, Rekha Pobbysetty, Suhel Hassan

**Affiliations:** ^1^Department of Radiology, Narayana Multispeciality Hospital and Mazumdar Shaw Medical Center, Narayana Health City, Bangalore, India; ^2^Department of Pathology, Narayana Multispeciality Hospital and Mazumdar Shaw Medical Center, Narayana Health City, Bangalore, India; ^3^Department of Otolaryngology, Narayana Multispeciality Hospital and Mazumdar Shaw Medical Center, Narayana Health City, Bangalore, India

## Abstract

Two cases of fibrous dysplasia involving the middle nasal turbinate are presented. Fibrous dysplasia is a common benign fibro-osseous disease involving the flat bones, often affecting the bony structures of the skull and facial skeleton. Primary occurrence or secondary involvement of the nasal turbinate is not a common manifestation of the disease. Involvement of the inferior turbinate generally does not have specific management-related issues; however, involvement of the middle turbinate, especially the lateral lamella, can predispose to surgical morbidity during endoscopic surgical management. Clinical presentation, management and features of the disease on CT imaging are presented.

## Background

Fibrous dysplasia (FD) is a condition characterized by progressive replacement of the normal bony structures with benign cellular fibrous connective tissue, leading to disorganized structure of the bone.^1^ Depending on the extent of involvement of the skeletal components, the disease can be categorized as monostotic (limited to a single bone) or polyostotic.^1–3^ The most common sites of involvement are the membranous bones of the pelvic girdle, femur and tibia. Skull is also a common site of involvement. Involvement of the nasal turbinate is infrequent.^3–5^ Isolated involvement of the inferior turbinate, in view of its anatomical relation, does not predispose to significant management problems. However, involvement of the middle and superior turbinate, and lateral and basal lamella creates a unique situation wherein there is involvement of the skull base structures, leading to increased surgical morbidity. Involvement of the lateral lamella, by virtue of its attachment to the cribriform plate and proximity to the ethmoidal arteries, predisposes to risk of damage to the floor of the anterior fossa and vascular injury during surgical or endoscopic intervention.

## Case reports

**Figure 1.**
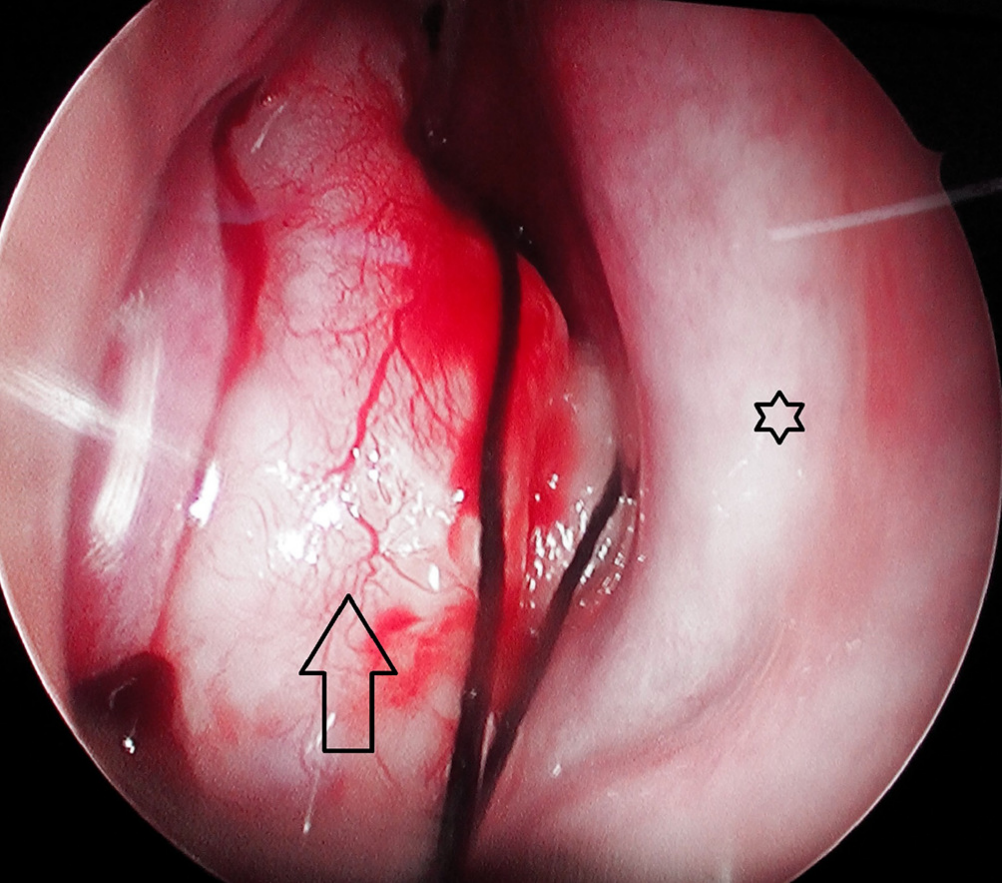
Anterior nasal endoscopic view of the right nasal cavity demonstrating a mass protruding through the anterior nasal cavity (arrow) in the anatomical space of the middle turbinate. The nasal septum is visualized medially (star).

**Figure 2.**
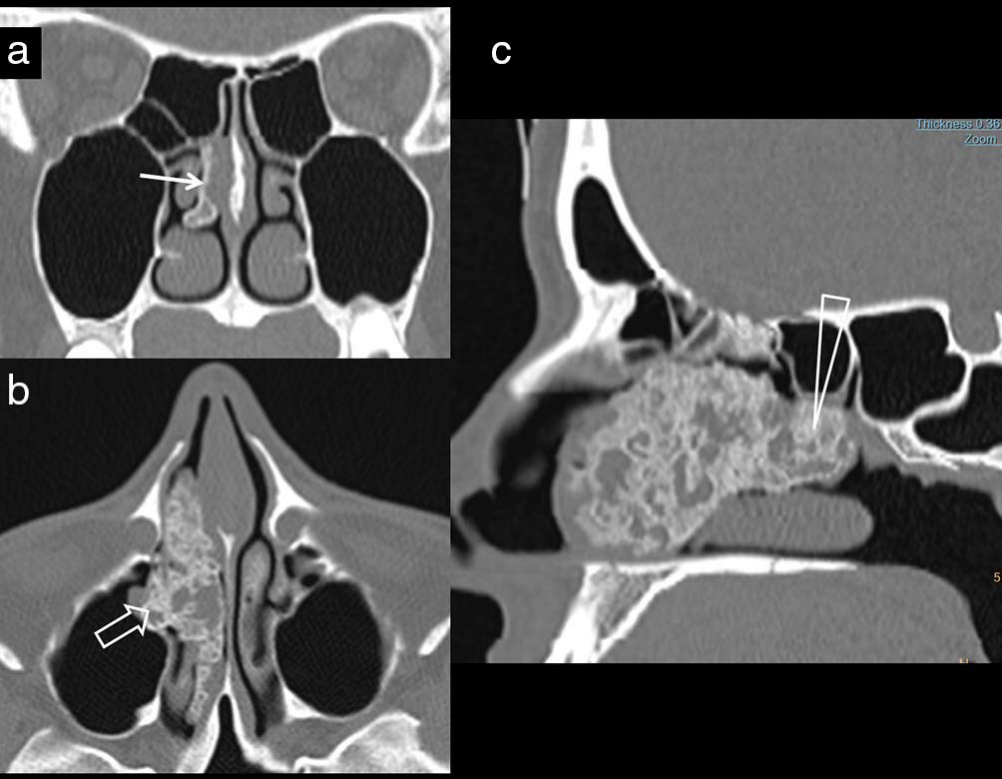
A 31-year-old female patient presenting with nasal block. (a) Non-contrast-enhanced coronal CT images demonstrate a minimally thickened middle turbinate (arrow). (b) The axial image demonstrates extensive areas of heterogeneous calcification in the mid-nasal cavity (open arrow), extending anteriorly to the right nasal vestibule. (c) Parasagittal CT reconstruction demonstrates the lesion extending to the basal lamella (triangle).

**Figure 3.**
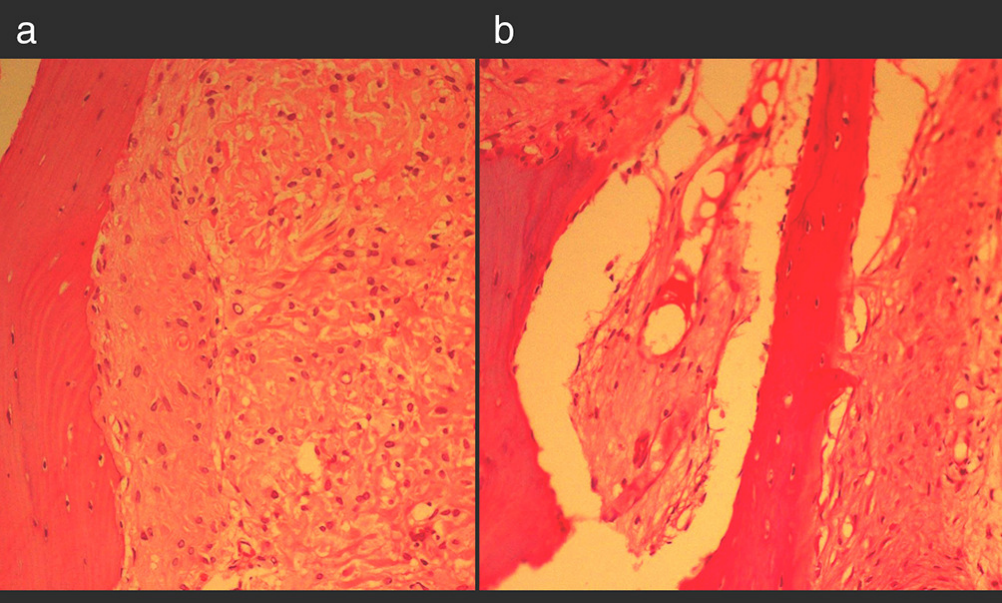
Haematoxylin and eosin-stained photomicrograph (a) shows isolated trabeculae of woven bone without osteoblastic rimming and intervening cellular fibrous tissue comprising benign spindle to polygonal cells with no atypical features. (b) Additional photograph demonstrates sheets of mature bone separated by focal cellular areas with formation of fibrous and hyaline cartilage nodules.

Case 1: a 31-year-old female patient was referred for imaging for nasal block. The patient had history of 6–8 months of recurrent epistaxis but no history of headache or any systemic complaints. Clinical examination demonstrated a hard mass protruding through the right nostril (Figure 1). Laboratory parameters, including levels of alkaline phosphatase, were normal. CT imaging of the nasal cavity was performed. Examination demonstrated enlargement of the posterior aspect of the basal lamella owing to a mass containing non-homogeneous areas of calcification. The lesion was occupying most of the mid-nasal cavity by displacing the inferior turbinate and extending into the nasal vestibule (Figure 2). The patient underwent endoscopic surgery under general anaesthesia and complete excision of the mass was performed. The mass was adherent to the posterior aspect of the septum and the medial surface of the inferior turbinate. The resected specimen consisted of pieces of bony fragments, with the largest component measuring 3 × 2 × 1 cm. Microscopic evaluation demonstrated features suggestive of a benign fibro-osseous lesion, favouring FD (Figure 3).

**Figure 4.**
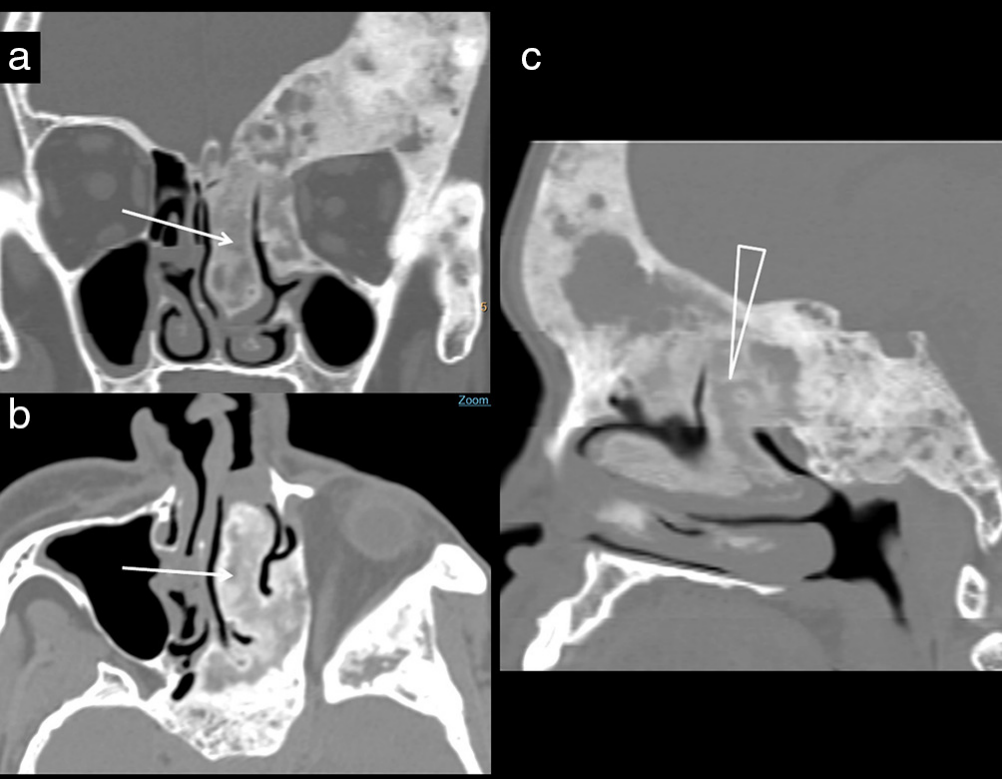
A 32-year-old adult female with occasional nasal bleeding. (a) Coronal CT image at the mid-nasal cavity level demonstrates thickening of the orbital plate and zygomatic bone. There is massive thickening of the lateral lamella and middle turbinate (arrow), encroaching upon the superior aspect of the left nasal cavity. (b) Axial image demonstrates an enlarged superior turbinate showing posterior continuity with the skull base (arrow), demonstrated better on the sagittal CT reconstruction (triangle; c).

Case 2: a 32-year-old adult female presented with occasional nasal bleeding, difficulty in breathing and recurrent headaches. She had no visual complaints. Clinical examination was unremarkable. Nasal endoscopy revealed obliteration of the superior aspect of the left nasal cavity, with poor visualization of the details. Multidetector CT evaluation of the nasal cavity and skull was performed. CT examination revealed gross sclerosis of the frontal bone, orbital plates, zygomatic bone, ethmoid and sphenoid. The involved bone showed gross thickening of the inner and outer tables with obliteration of the normal architecture. There was uneven and disorganized structure of the skull bones involving the squamous part, orbital plate of the frontal bone, medial ethmoid, zygomatic bone and sphenoid. There was gross homogeneous enlargement of the lateral and basal lamella of the middle turbinate (Figure 4). The whole length of the turbinate was involved, with ground-glass texture and preserved overall configuration. Owing to increase in the thickness of the turbinate and additional involvement of the medial wall of the ethmoidal sinus, the nasal cavity was completely occluded. There was significant narrowing of the superior orbital fissure. The optic canal and optic foramina were not obliterated. After due consideration of the cost and benefits of surgical intervention, the patient was advised to have regular check-ups. Surgical option was deferred for a later date, subject to progression of symptoms.

## Discussion

FD is a skeletal disorder constituting 7.5% of the benign neoplastic bone lesions.^1,2^ It can present as involving a single bone (monostotic: 75–80%) or multiple bones (polyostotic: 20–25%).^2^ Common sites of single bone involvement are the ribs, and femur and tibia, and it equally affects both males and females. The polyostotic form has varied presentation with a female predilection. Facial bone involvement is more frequent in the polyostotic form and typically presents at around 10 years of age.^2^ Frequent sites of craniofacial FD are the maxilla; mandible; and frontal, parietal and occipital bones. The exact incidence of involvement of the nasal cavity is unknown. Although most cases of facial involvement are asymptomatic and found incidentally, many with involvement of nasal cavity are symptomatic. Clinical presentation of the nasal cavity lesion often manifests as encroachment, distortion of anatomic spaces and mass effect. Clinical features are nasal obstruction, bleeding, headache, proptosis, diplopia, anosmia, epistaxis and recurrent rhinosinusitis.^5^

The pathological feature of FD is inability of the bone-forming tissue to produce mature lamellar bone. Examination of the bone shows accumulation of arrested tissue consisting of immature woven bone. Molecular studies have suggested that FD is a genetic non-inherited condition caused by missense sporadic mutation of the gene *GNAS1* on chromosome 20, which encodes the alpha subunit of the stimulatory G protein-coupled receptor.^6^ Hence, organs that have the stimulatory G protein-coupled receptors, such as bone, skin, ovaries, thyroid and pituitary glands, are often affected. Therefore, the clinical spectrum of FD is variable, depending upon the stage of embryogenesis when mutation had occurred.

Imaging appearances of FD are variable and depend upon the site of involvement and proportion of “mineralized bone to fibrous tissue” in the lesion.^1,6^ Typical imaging description of FD categorizes lesions into the following forms: ground-glass pattern (56%), uniformly sclerotic pattern (23%) and cystic variety (21%).^7^ Plain radiography is typically used as an initial step and often suggests the diagnosis in the large majority of patients. However, specific anatomical details may not be obtainable by plain radiography. Ground-glass appearance of the involved bone, with obliteration of corticomedullary distinction, is highly characteristic of FD on plain radiography as well as cross-sectional imaging. The sclerotic form of the disease can be mistaken for an osteoma or an ossifying fibroma. The cystic variety and mixed form of FD may have non-specific imaging features. CT scan and MRI are useful in detecting involvement of specific subsites, defining specific landmarks and demonstrating pressure on the neurovascular bundle, occlusion of the ostia and involvement of the cribriform plate and optic apparatus. CT appearances can range from uniformly sclerotic bone changes to focal osteolytic process, depending on the degree of mineralization of the tissue.^1^ Classically, on MRI, FD shows hypointensity in *T*_1_ and *T*_2_ weighted sequences, owing to a large fibrous component of the lesion. However, signal intensity of the bone lesions can vary depending on the composition of the bone and extent of mineralization.^6^ Bone scan can be utilized for identifying polyostotic disease, which is shown as multiple areas of increased radioisotope uptake.^6^ Whole-body MRI has an important role in assessing the extent of the other sites of involvement without ionizing radiation in the polyostotic form of disease.

In atypical cases, histological evaluation becomes critical. On gross examination, the lesions consist of fibrous tissue and show varying consistency and vascularity.^6^ Microscopically, the lesion shows irregular trabeculae of woven bone blending into the surrounding normal bone and lying within a cellular fibrous stroma.^1,6^ Histological appearances match the stages of FD. In the acute stage, there is rich cellular connective tissue with mitotic figures and woven immature bone, whereas tissues in the subacute stage become less cellular and more fibrous, and the fibres tend to be arranged in whorls. The bony trabeculae become thicker and show lamination. In the chronic stage, laminated bony trabeculae occur in abundance with a rim of osteoblasts.^8^ Total serum bone alkaline phosphatase and urinary hydroxyproline are elevated in the active phase of FD in approximately 75% of patients.^6^ Major differential diagnoses of FD of the nasal cavity are ossifying fibroma and lesions of chondrogenic origin.

Asymptomatic, stable lesions that are located in a silent area, not causing deformities or functional impairment, can be followed up.^1,6^ Symptomatic nasal cavity lesions often need surgical management. Precise location and growth potential of the lesion decide the exact nature of the surgery to be performed; imaging information appears to be more accurate in delineating the origin of the lesion in comparison with nasal endoscopy. The epicentre of the lesion, location of predominant bulk and extent of involvement of the adjacent structures dictate the surgical approach. Our first patient had nasal obstruction owing to the lesion arising from the posterior part of the middle turbinate and centred on the mid and lower nasal cavity without significant involvement of adjacent structures. Thus, the lesion was well suited for transnasal endoscopic approach. Considering the patient’s age and symptomatic presentation, the tumour was removed with endoscopic endonasal approach. In the second patient, the lesion was located in the superior aspect of the nasal cavity, with the lesion being part of the more generalized changes of FD involving the frontal bone and ethmoidal sinus. Moreover, the lesion involved the lateral lamella and its attachment with the cribriform plate. Attempted surgical or endoscopic removal involving the lateral/basal lamella in this patient had the potential risk of causing damage to the cribriform plate. In view of the total extent of the disease and the relatively minor clinical symptoms, the patient was managed conservatively. Open surgical approaches, including the midfacial degloving, lateral rhinotomy, transantral approach and the Le Fort I osteotomy, may be employed in large, inaccessible lesions with broad attachment to the surrounding structures.^9^

Regional anatomy plays an important role in the management of FD involving the middle turbinate. Configuration of the ethmoidal roof and depth of the cribriform plate on CT scan have been classified by Keros into three anatomical categories. Category 3, which is associated with deep olfactory fossa, is the most vulnerable to iatrogenic damage during surgery owing to a long lateral lamella and close vicinity of the anterior fossa structures to the nasal cavity. In addition, the point of entry of the anterior ethmoidal artery is thinnest in the region, predisposing to cerebrospinal fluid leak and vascular damage leading to haemorrhage and orbital haematoma.^10–13^ Spontaneous sarcomatous transformation of FD is very rare, less than 1%, with most patients having received radiation therapy.^8^ The prognosis for FD is generally good, although outcomes are poorer in young patients and those with the polyostotic forms.^6^ Recurrence is rare in adults, but the lesions can show unexpected growth potential if they are surgically altered during their active growth phase.

## Learning points

1. FD of the craniofacial bones is common in occurrence, but involvment of the nasal turbinate, either in isolation or along with the adjacent bones, is very infrequent.
2. Involvement of the middle turbinate, especially the lateral lamella, has clinical significance, as it poses a risk of damage to the cribriform plate and skull base during surgical or endoscopic intervention.
3. Additional risk involves possible injury to the ethmoidal arteries, which pass through the respective foramina of the involved bone.
4. Multidetector CT imaging with soft tissue and bone reconstruction and evaluation of the region in multiple planes provides comprehensive diagnosis.

## Consent

Informed consent was obtained for publication of images and clinical photographs.

## References

[1] Feller L, Wood NH, Khammissa RA, Lemmer J, Raubenheimer EJ The nature of fibrous dysplasia. Head Face Med 2009; 5: 22. 19895712 10.1186/1746-160X-5-22 PMC2779176

[2] Sadeghi SM, Hosseini SN Spontaneous conversion of fibrous dysplasia into osteosarcoma. J Craniofac Surg 2011; 22: 959–61. 21558913 10.1097/SCS.0b013e31820fe2bd

[3] Park HJ, Cho MS, Lee SS Fibrous dysplasia of the inferior turbinate. Int J Clin Exp Pathol 2013; 6: 531–5 23411641 PMC3563202

[4] Karligkiotis A, Terranova P, Dallan I, Castelnuovo P Monostotic fibrous dysplasia of the inferior turbinate. Otolaryngol Head Neck Surg 2012; 146: 1035–6. 22157265 10.1177/0194599811431059

[5] Ozcan KM, Akdogan O, Gedikli Y, Ozcan I, Dere H, Unal T Fibrous dysplasia of inferior turbinate, middle turbinate, and frontal sinus. B-ENT 2007; 3: 35–8 17451125

[6] Parekh SG, Donthineni-Rao R, Ricchetti E, Lackman RD Fibrous dysplasia. J Am Acad Orthop Surg 2004; 12: 305–13. 15469225 10.5435/00124635-200409000-00005

[7] Lisle DA, Monsour PA, Maskiell CD Imaging of craniofacial fibrous dysplasia. J Med Imaging Radiat Oncol 2008; 52: 325–32. 18811755 10.1111/j.1440-1673.2008.01963.x

[8] DiCaprio MR, Enneking WF Fibrous dysplasia. Pathophysiology, evaluation, and treatment. J Bone Joint Surg Am 2005; 87: 1848–64. 16085630 10.2106/JBJS.D.02942

[9] Archontaki M, Stamou AK, Hajiioannou JK, Kalomenopoulou M, Korkolis DP, Kyrmizakis DE Cavernous haemangioma of the left nasal cavity. Acta Otorhinolaryngol Ital 2008; 28: 309–11 19205597 PMC2689539

[10] Nair S Importance of ethmoidal roof in endoscopic sinus surgery. Sci Rep 2012; 1: 251.

[11] Stammberger H Endoscopic anatomy of lateral wall and ethmoidal sinuses Stammberger H, Hawke M, eds Essentials of functional endoscopic sinus surgery. St Louis, MO: Mosby-Year Book; 1993 pp. 13–42

[12] Başak S, Karaman CZ, Akdilli A, Mutlu C, Odabaşi O, Erpek G, et al Evaluation of some important anatomical variations and dangerous areas of the paranasal sinuses by CT for safer endonasal surgery. Rhinology 1998; 36: 162–7 9923058

[13] Hwang PH, Abdalkhani A Embryology, anatomy and physiology of the nose and paranasal sinuses In: Snow JB, Wackym PA, Ballenger JJ Ballenger’s otorhinolaryngology: head and neck surgery. Revised edition Shelton, CT: PMPH-USA; 2009 pp. 455–63

